# Two new species of *Neopestalotiopsis* from southern China

**DOI:** 10.3897/BDJ.9.e70446

**Published:** 2021-08-25

**Authors:** Qi Yang, Xiang-Yu Zeng, Jun Yuan, Qian Zhang, Yu-Ke He, Yong Wang

**Affiliations:** 1 Department of Plant Pathology, Agricultural College, Guizhou University, Guiyang, China Department of Plant Pathology, Agricultural College, Guizhou University Guiyang China; 2 Center of Excellence in Fungal Research, Mae Fah Luang University, Chiang Rai, Thailand Center of Excellence in Fungal Research, Mae Fah Luang University Chiang Rai Thailand

**Keywords:** two new taxa, Sporocadaceae, taxonomy

## Abstract

**Background:**

Pestalotiopsis-like fungi are widely distributed in many plants and include endophytes, pathogens and saprobes. Five strains of *Neopestalotiopsis* were isolated from diseased leaves of *Rhapisexcelsa* (Principes, Palmae), *Rhododendronsimsii* and *Rho.championiae* (Ericales, Ericaceae) and *Erythropalumscandens* (Santalales, Olacaceae) in southern China.

**New information:**

Based on morphology and multi-gene (ITS, *tub2*, *tef1*) phylogeny, our five strains of *Neopestalotiopsis* represent two new species and one extant species. Descriptions, illustrations and notes are also provided for the new species.

## Introduction

Sporocadaceae was introduced by Corda (1842) and comprised abundant endophytic, plant pathogenic or saprobic taxa (Liu et al. 2019). A great part of Sporocadaceae species were reported as important plant pathogenic fungi that mainly harm various economic crops, such as tea, blueberry and elephant apple (Fernández et al. 2015, Banerjee et al. 2018, Tsai et al. 2020). Jaklitsch et al. (2016) synonymised Bartaliniaceae, Discosiaceae, Pestalotiopsidaceae and Robillardaceae under Sporocadaceae. Liu et al. (2019) studied the taxonomy of Sporocadaceae and accommodated 30 genera in it. Hyde et al. (2020) and Wijayawardene et al. (2020) placed Sporocadaceae in Amphisphaeriales and accepted 33 genera.

*Neopestalotiopsis* was introduced by Maharachchikumbura et al. (2014) to accommodate pestalotiopsis-like taxa that had versicolorous median cells and indistinct conidiophores. Until now, 49 taxa of *Neopestalotiopsis* are known (Mycobank 2021: https://www.mycobank.org/page/Home). This group commonly occurs on plants as endophytes, pathogens or saprobes (Jeewon et al. 2004, Liu et al. 2010, Hyde et al. 2016, Reddy et al. 2016, Shetty et al. 2016, Ran et al. 2017, Bezerra et al. 2018, Freitas et al. 2019). Recently, research showed them as plant pathogens causing stem blight, flower bight, twig dieback and fruit rot (Akinsanmi et al. 2016, Borrero et al. 2017, Mahapatra et al. 2018, Rodríguez-Gálvez et al. 2020). In the past few years, China and Thailand are places where most species of *Neopestalotiopsis* were found (Norphanphoun et al. 2019).

Amongst surveys of microfungi in southern China, we made five collections of *Neopestalotiopsis* from four host plants. Based on morphological descriptions and molecular analyses of three gene loci, our strains represent two new species and one known species.

## Materials and methods

**Sample collection and fungi isolation**

Diseased leaf samples with fruiting bodies were collected from major botanical gardens in Yunnan, Guangxi and Guizhou Provinces in southern China. After surface disinfection of the diseased tissues (Zhang et al. 2020), the single-spore method was used for obtaining a pure culture (Senanayake et al. 2020). The isolates were transferred to new potato dextrose agar (PDA) plates to obtain a pure strain.

**Morphology study**

Cultures growing on potato dextrose agar (PDA) were incubated under moderate temperatures (28ºC) for 2−4 weeks in 12 h daylight. The diameter of cultures was measured after 1 week and the colour was determined with the colour charts of Rayner (1970). The morphological features were noted and recorded following Hu et al. (2007). Microscopic preparations were prepared in lactophenol and over 30 measurements were obtained per structure. Photographs were taken using a compound microscope (Olympus BX53, Japan). The holotype specimens were deposited in the Herbarium of Department of Plant Pathology, Agricultural College, Guizhou University (HGUP). Ex-type cultures were deposited in the Culture Collection at the Department of Plant Pathology, Agriculture College, Guizhou University, China (GUCC).

**DNA extraction and PCR amplification**

DNA extraction and PCR amplification follow Dissanayake et al. (2020) with some minor changes. A Fungus Genomic DNA Extraction Kit (Biomiga#GD2416, San Diego, California, USA) was used to extract fungal genome DNA. DNA amplification was performed in a 25 µl reaction mixture which contains 2.5 µl 10 × PCR buffer, 1 µl of each primer (10 µM), 1 µl template DNA and 0.25 µl Taq DNA polymerase (Promega, Madison, WI, USA). The ITS rDNA region was amplified using primer pairs ITS4 and ITS5 (White et al. 1990). The partial *tub2* gene region was amplified with primer pairs T1 and Bt2b (Glass and Donaldson 1995, O'Donnell and Cigelnik 1997). The *tef1* gene fragment was amplified using the primer pairs EF1-728F and EF-2 (O'Donnell et al. 1998, Carbone and Kohn 1999). PCR amplification conditions were performed according to the methods described by Norphanphoun et al. (2019). The PCR products were sent to SinoGenoMax company (Beijing, China) which used the fluorescently-labelled Sanger method for sequencing. The resulting DNA sequences were submitted to GenBank (https://www.ncbi.nlm.nih.gov/genbank/) and their accession numbers were provided in Table 1.

**Sequence alignment and phylogenetic analyses**

The reference sequences were downloaded from GenBank for phylogenetic analyses (Table 1). Multiple sequence alignments were generated with MAFFT v. 7.307 online version (Katoh and Standley 2016) and manually improved in MEGA v. 6.06, where necessary (Tamura et al. 2013). Concatenated multi-locus datasets for the three gene regions were aligned using Mesquite v. 2.75 (Maddison 2008). Manual improvement, when necessary, was done using AliView (Larsson 2014). Terminal ends and ambiguous regions of the alignment were deleted manually. Phylogenetic analyses were performed using concatenated sequences of the three loci (ITS, *tub2* and *tef1*) with Maximum Likelihood (ML), Maximum Parsimony (MP) and Bayesian Inference (BI).

Maximum Likelihood analysis was performed at the CIPRES Science Gateway web portal (Miller et al. 2010) using RAxML-HPC BlackBox v. 8.2.12 with the GTR+G+I model and 1,000 rapid bootstrap (BS) replicates (Stamatakis 2014).

Bayesian analysis was conducted with MrBayes v. 3.1.2 (Huelsenbeck and Ronqvist 2001). Parameters of Bayesian analysis in MrBayes v. 3.2; Markov chains were run for 1000000 generations and trees were sampled every 100th generation (printfreq = 100) and 10000 trees were obtained. The last standard deviation of split frequencies was below 0.01. Initial trees were discarded (25% burn-in value) and the remaining trees were used to evaluate posterior probabilities (PP) in the majority rule consensus tree.

PAUP v. 4.0b10 (Swofford 2002) was used to perform Maximum Parsimony (MP) analyses. Trees were inferred by using the heuristic search option with 1,000 random sequence additions and tree bisection and reconnection (TBR) as the branch-swapping algorithm. The maxtrees were set as 5000. Descriptive tree statistics for parsimony (tree length (TL), consistency index (CI), retention index (RI), related consistency index (RC) and homoplasy index (HI)) were calculated.

## Taxon treatments

### Neopestalotiopsis rhapidis

Qi Yang & Yong Wang bis sp. nov.

1EC88425-ADBF-5527-B845-9EF7658D0BA9

840065

#### Materials

- **Type status:**Holotype. **Occurrence:** recordedBy: Qi Yang; occurrenceID: GUCC 21501; **Taxon:** scientificName: *Neopestalotiopsisrhapidis*; order: Amphisphaeriales; family: Sporocadaceae; genus: Neopestalotiopsis; **Location:** country: China; stateProvince: Guangxi; locality: Nanning City, Guangxi Medicinal Botanical Garden; verbatimCoordinates: 108°19’ E,22°51’ N; **Identification:** identifiedBy: Qi Yang; dateIdentified: 2021; **Record Level:** collectionID: HGUP 332

#### Description

**Disease symptom**: Pathogenic causing spots on leaves tip of *Rhapisexcelsa*. Leaf spots shape irregular, brown, slightly sunken on leaves tip. Small brown spots appeared initially and then gradually enlarged, changing to dark brown spots with a yellow border and jagged edge.

**Figure 1.**
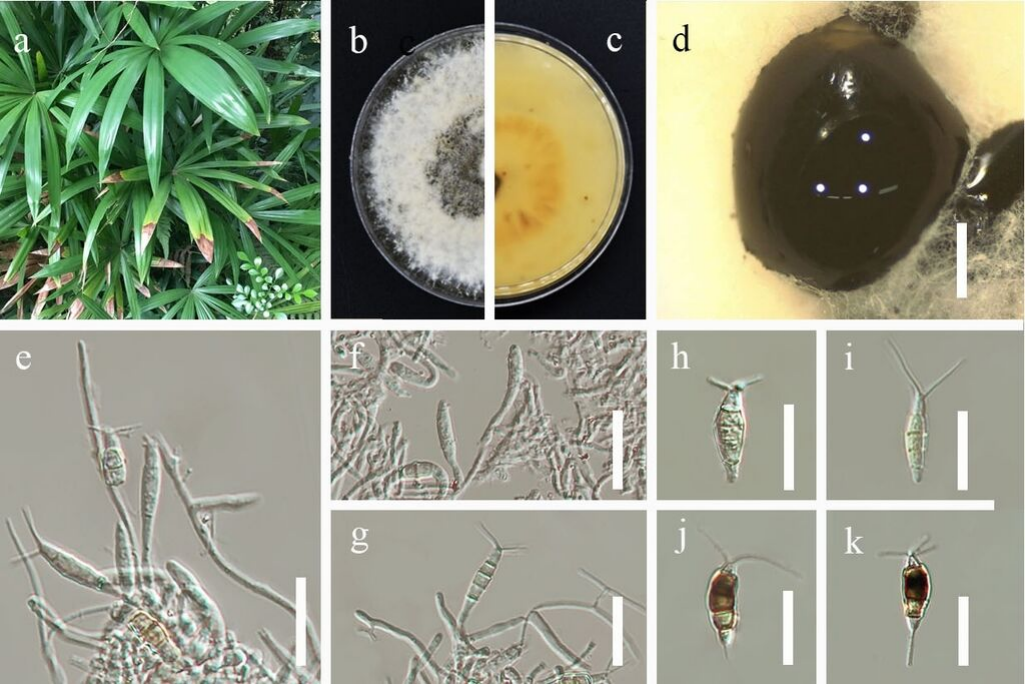
*Neopestalotiopsisrhapidis* (GUCC 21501). **a.** Leaf spots of *Neopestalotiopsisrhapidis*; **b, c.** Culture on PDA (**b**-above, **c**-reverse); **d.** Colony sporulating on PDA; **e–g.** Conidiophores; **h–k.** Conidia. Scale bars: **d** = 1000 µm, **e–k** = 20 µm.

*Colonies* on PDA reach 7.5–8 cm in diam. after 7 d at room temperature (28°C), under light 12 hr/dark. *Colonies* filamentous to circular, whitish, with clustered black fruiting bodies and filiform and fluffy margin, white from above and light yellow from the reverse. Sexual morph: undetermined. Asexual morph（Fig. 1): *Conidiomata* 560–1405 µm in diam., pycnidial, globose, solitary, black, semi-immersed on PDA, exuding brown to dark brown conidia. *Conidiophores* branched or unbranched, hyaline, thin-walled. *Conidiogenous cell* discrete to lageniform, obclavate, hyaline or rarely light brown, smooth-walled. *Conidia* (22–)25.5 × 4(–6) µm (x̄ = 23 × 5.2 µm, n = 30), fusiform to clavate, straight to slightly curved, 4-septate; basal cell cylindrical to obconic, hyaline, thin-walled, smooth, 3–5 µm (x̄ = 3.7 µm, n = 30); the three median cells 11.5–15 µm (x̄ = 13.3 µm, n = 30), dark brown with septa darker than the rest of the cells, the second cell from base 3–5 µm (x̄ = 4 µm, n = 30); the third cell 2.5–6 µm (x̄ = 3.9 µm, n = 30); the fourth cell 3–4.5 µm (x̄ = 3.8 µm, n = 30); apical cell 2–4.5 µm (x̄ = 3.3 µm, n = 30), cylindrical, hyaline; 2–3 (mostly 3) tubular apical appendages, arising from the apex of the apical cell each at different points, flexuous, 11–16 µm (x̄ = 13.6 µm, n = 30); basal appendage present, single, tubular, unbranched, 2–5.5 µm (x̄ = 4 µm, n = 30).

#### Etymology

Latin, *rhapidis*, refers to the host plant (*Rhapisexcelsa*) from which the fungus was isolated.

#### Notes

*Neopestalotiopsisrhapidis* clustered with *N.cocoes* (MFLUCC 15-0152) with 85% ML support, although without enough MP and BI support. Within comparison of the three gene regions, there were only three character differences in the ITS region, but 27 in the *tef1* region. *Neopestalotiopsisrhapidis* has longer conidia and shorter apical appendages than those of *N.cocoes* (19–22.5 ×7.5–9.5 µm; 14.9–21 µm) (Hyde et al. 2016). Thus, *Neopestalotiopsisrhapidis* (GUCC 21501) is introduced as a new species herein.

### Neopestalotiopsis rhododendri

Qi Yang & Yong Wang bis sp. nov.

A0E690E4-DC7E-5D2F-A228-0FCD3E9966DB

840066

#### Materials

- **Type status:**Holotype. **Occurrence:** recordedBy: Qi Yang; occurrenceID: GUCC 21504; **Taxon:** scientificName: *Neopestalotiopsisrhododendri*; order: Amphisphaeriales; family: Sporocadaceae; genus: Neopestalotiopsis; **Location:** country: China; stateProvince: Yunnan; locality: Kunming; verbatimCoordinates: 102°72’ E,25°05’ N; **Identification:** identifiedBy: Qi Yang; dateIdentified: 2021; **Record Level:** collectionID: HGUP 134
- **Type status:**Other material. **Occurrence:** recordedBy: Qi Yang; occurrenceID: GUCC 21505; **Taxon:** scientificName: *Neopestalotiopsisrhododendri*; order: Amphisphaeriales; family: Sporocadaceae; genus: Neopestalotiopsis; **Location:** country: China; stateProvince: Guizhou; locality: Kaili; verbatimCoordinates: 107°97’ E,26°58’ N; **Identification:** identifiedBy: Qi Yang; dateIdentified: 2021; **Record Level:** collectionID: HGUP 997

#### Description

**Disease symptom**: Associated with leaf spots of *Rhododendronsimsii*. The leaf spots are small irregular to subcircular shape, brown, slightly sunken spots appear on surface leaves of *R.simsii*, which scattered on the surface leaves tip and eventually develops into a large lesion. Small off-white spots appeared initially and then gradually enlarged, changing to light brown circular ring spots with a dark brown border.

**Figure 2.**
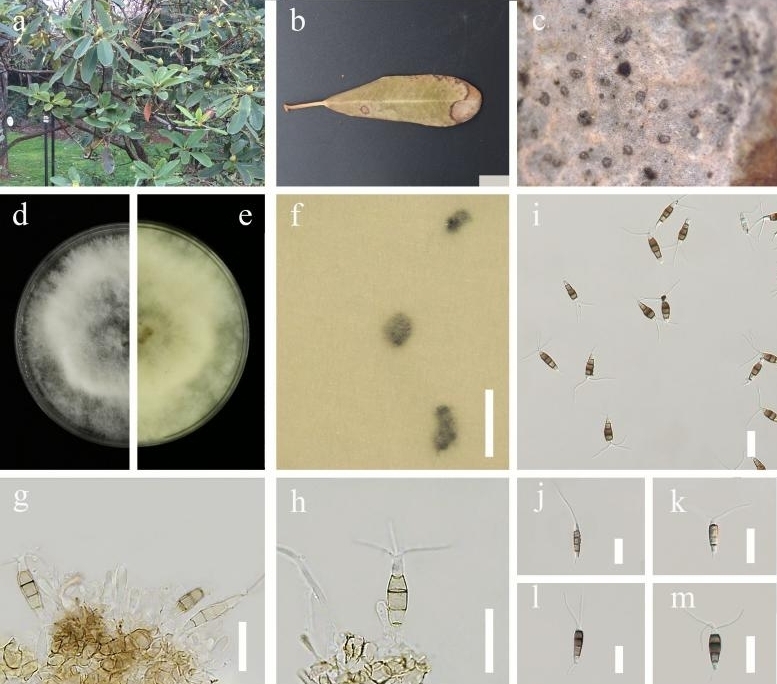
*Neopestalotiopsisrhododendri* (GUCC 21504). **a, b, c.** Leaf spots of *Neopestalotiopsisrhododendri*; **d, e.** Culture on PDA (**d**-above, **e**-reverse); **f.** Colony sporulating on PDA; **g–h.** Conidia and conidiophores; **i–m.** Conidia. Scale bars: **f** = 1000 µm, **g–m** = 20 µm.

*Colonies* on PDA reaching 6.5–7 cm in diam. after 7 d at room temperature (28°C), under light 12 hr/dark. *Hyphae* white, colonies filamentous to circular, slightly undulate at the edge, with black fruiting bodies clustered, has filiform and fluffy margin, white from above and light yellow from the reverse. Sexual morph: undetermined. Asexual morph（Fig. 2）: *Conidiomata* 55–280 µm in diam., pycnidial, globose, solitary, black, semi-immersed on PDA, exuding brown to dark brown mass of conidia. *Conidiophores* often reduced to conidiogenous cell, regularly septate and branched at the base. *Conidiogenous cells* mostly integrated, ampulliform, cylindrical, hyaline to light brown, smooth-walled. *Conidia* (25.5–)30 × 5(–6) µm (x̄ = 27.6 × 5.5 µm, n = 30), fusiform to clavate, straight to slightly curved, 4-septate; basal cell obconic, hyaline, thin-walled, smooth, 3.5–6.5 µm (x̄ = 4.5 µm, n = 30); the three median cells 13.5–19.5 µm (x̄ = 16.3 µm, n = 30), light brown to dark brown, dark brown with septa darker than the rest of the cells, the second cell from base 4–6 µm (x̄ = 5 µm, n = 30); the third cell 3.5–5.5 µm (x̄ = 4.5 µm, n = 30); the fourth cell 4–6.5 µm (x̄ = 4.8 µm, n = 30); apical cell 3.5–6.3 µm (x̄ = 5 µm, n = 30), cylindrical to sub-cylindrical, hyaline, 1–3 (mostly 2) tubular apical appendages, arising from the apex of the apical cell each at different points, 21–38.5 µm (x̄ = 29.2 µm, n = 30); basal appendage present most of the time, single, tubular, unbranched, 6–11.5 µm (x̄ = 8.5 µm, n = 30).

#### Etymology

China, Yunnan Province, Kunming City, from leaves of *Rhododendronsimsii*, 12 February 2018, Q. Zhang, HGUP 134, holotype, ex-type living culture GUCC 21504.

#### Notes

In the multi-gene analysis, strain GUCC 21504 formed a distinct clade with a sister strain GUCC 21505, but the node support values were 68/90/- (MP/ML/BI) and these two strains were close to *N.protearum* (CBS 114178). When comparing the polymorphic nucleotide differences of our two strains, there are 18 base pair differences, seven in ITS, two in *tub2* and nine in *tef1*, but without obvious distinction (higher than 98.5%). Compared with *N.protearum* and our ex-type strain (GUCC 21504), there were six character differences with *N.protearum* in the ITS region, three character differences with *N.protearum* in the *tub2* region, but 12 character differences from *N.protearum* in the *tef1* region; thus the DNA base pair differences were mainly in the *tef1* gene regions. The morphological differences between our strains and *N.protearum* were wider conidia (*N.protearum*: 24.8 ± 1.5 × 8.5 ± 0.6 µm), more apical appendages (*N.protearum*: 3–5) and shorter basal appendages (*N.protearum*: 5–8 µm) (Maharachchikumbura et al. 2014). Thus, *Neopestalotiopsisrhododendri* is introduced as a novel taxon, based on morphology and phylogeny.

### Neopestalotiopsis saprophytica

(Maharachch. & K.D. Hyde) Maharachch., K.D. Hyde & Crous, 2014

ADF06C5C-4E31-55DF-BDC6-3237DE7E2CCF

809780

#### Materials

- **Type status:**Other material. **Occurrence:** recordedBy: Qi Yang; occurrenceID: GUCC 21506; **Taxon:** scientificName: *Neopestalotiopsissaprophytica*; order: Amphisphaeriales; family: Sporocadaceae; genus: Neopestalotiopsis; **Location:** country: China; stateProvince: Guangxi; locality: Nanning City，Guangxi Medicinal Botanical Garden; verbatimCoordinates: 108°19’ E,22°51’ N; **Identification:** identifiedBy: Qi Yang; dateIdentified: 2021; **Record Level:** collectionID: HGUP 423
- **Type status:**Other material. **Occurrence:** recordedBy: Qi Yang; occurrenceID: GUCC 21507; **Taxon:** scientificName: *Neopestalotiopsissaprophytica*; order: Amphisphaeriales; family: Sporocadaceae; genus: Neopestalotiopsis; **Location:** country: China; stateProvince: Guangxi; locality: Nanning City,Guangxi Medicinal Botanical Garden; verbatimCoordinates: 108°19’ E,22°51’ N; **Identification:** identifiedBy: Qi Yang; dateIdentified: 2021; **Record Level:** collectionID: HGUP 133

#### Description

**Disease symptom**: Pathogenic causing spots on leaves of *Erythropalumscandens*. Leaf spots shape irregular, brown to reddish-brown, slightly sunken spots appear on surface leaves of *E.scandens*, which scattered on the leaves tip. Small brown spots appeared initially and then gradually enlarged, changing to reddish-brown spots with a yellow border.

**Figure 3.**
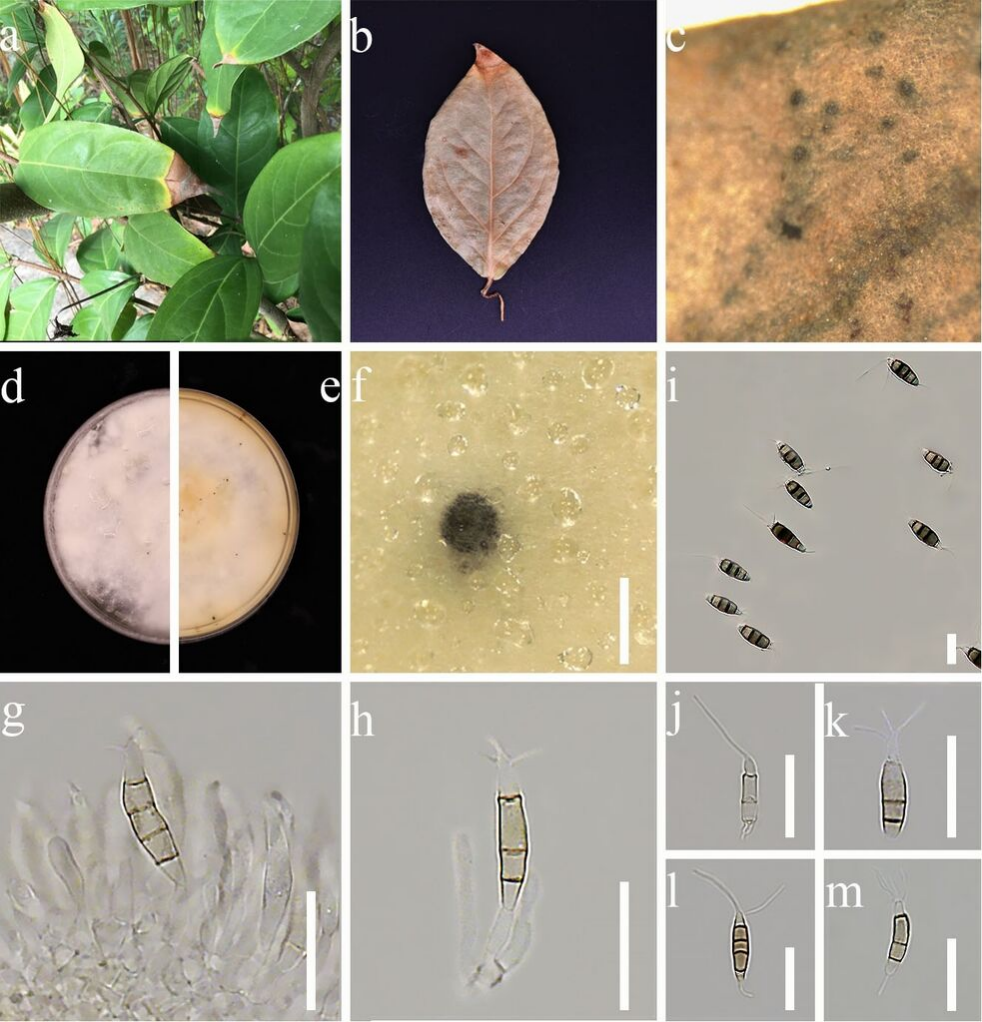
*Neopestalotiopsissaprophytica* (GUCC 21506). **a, b, c.** Leaf spots of *Neopestalotiopsissaprophytica*; **d, e.** Culture on PDA (**d**-above, **e**-reverse); **f.** Colony sporulating on PDA; **g–h.** Conidia and conidiophores; **i–m.** Conidia. Scale bars: **f** = 1000 µm, **g–m** = 20 µm.

*Colonies* on PDA reaching 7.5–8 cm in diam. after 7 d at room temperature (28℃), under light 12 hr/dark. *Hyphae* change from light pink to off-white. *Colonies* filamentous to circular, slightly undulate at the edge, with black fruiting bodies clustered, filiform margin, light pink from above and light yellow from the reverse. Sexual morph: undetermined. Asexual morph (Fig. 3): *Conidiomata* up to 280 μm in diam., pycnidial, globose, solitary, black, semi-immersed on PDA, exuding brown to dark brown mass of conidia. *Conidiophores* branched or unbranched, hyaline, thin-walled. *Conidiogenous cells* discrete, ampulliform to lageniform, hyaline, thin-walled, smooth. *Conidia* (21.5–)26.5 × 4.5(–6.5) µm (x̄ = 23.2 × 5.2 µm, n = 30), fusiform to clavate, straight to slightly curved, 4-septate; basal cell obconic, hyaline or sometimes pale brown, thin-walled, smooth, 3–5 µm (x̄ = 4 µm, n = 30); the three median cells 13–17 µm (x̄ = 14.9 µm, n = 30), pale brown to brown, dark brown with septa darker than the rest of the cells, the second cell from base 4–6.5 µm (x̄ = 4.9 µm, n = 30); the third cell 3–5 µm (x̄ = 4.1 µm, n = 30); the fourth cell 3.5–6 µm (x̄ = 4.8 µm, n = 30); apical cell 3–5 µm (x̄ = 3.9 µm, n = 30), cylindrical, hyaline; 1–4 (mostly 3) tubular apical appendages, arising from the apex of the apical cell each at different point, flexuous, 18–28.5 µm (x̄ = 22.4 µm, n = 30); basal appendage present most of the time, single, tubular, unbranched, 3.3–7 µm (x̄ = 4.3 µm, n = 30).

#### Notes

GUCC 21506 and GUCC 21507 with the same nucleotides sequences were related to *N.dendrobii* (MFLUCC 14-0106) and *N.saprophytica* (CBS 115452). There were ten character differences with *N.dendrobii* and 11 character differences with *N.saprophytica*, but the most differences (nine character differences) between our strains and *N.saprophytica* were only in the *tef1* region. Alternatively, collection differed to *N.dendrobii* in having more apical appendages (*N.dendrobii*: 2–3) and much longer apical appendages (*N.dendrobii*: 6 ± 0.9 µm) (Ma et al. 2019). Morphological characters of our collections and *N.saprophytica* overlapped (Maharachchikumbura et al. 2014). Thus, GUCC 21506 and GUCC 21507 are considered as *N.saprophytica*.

## Analysis

**Phylogenetic analyses**

**Figure 4.**
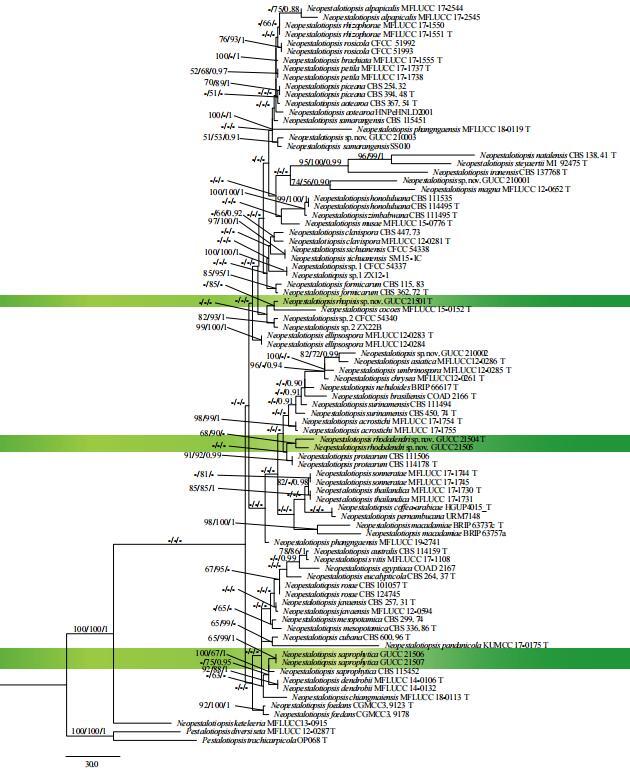
The phylogram generated from MP analysis, based on combined ITS, *tub2* and *tef1* sequence data of *Neopestalotiopsis*. The tree was rooted with *Pestalotiopsisdiversiseta* (MFLUCC 12-0287) and *P.trachicarpicola* (OP068). Maximum Parsimony and Maximum Likelihood bootstrap values ≥ 50%, Bayesian posterior probabilities ≥ 0.90 (MPBS/MLBS/PPBY) were given at the nodes. Our strains in this study were in green. Ex-type strains were marked by T.

The final dataset consists of 57 taxa, including *Pestalotiopsisdiversiseta* (MFLUCC 12-0287) and *P.trachicarpicola* (OP068) as the outgroup taxa. It comprised 2052 characters including gaps (*tef1*: 1−606, *tub2*: 607−1443 and ITS: 1444−2052). There were 1426 constant, 284 parsimony uninformative and 342 parsimony informative characters (TL = 1225 steps, CI = 0.66, RI = 0.70, RC= 0.46 and HI= 0.34). The most parsimonious tree generated from combined ITS, *tub2* and *tef1* sequence data of species of *Neopestalotiopsis* is shown in Fig. 4.

In the phylogenetic analyses, GUCC 21501 was sister to *N.cocoes* (MFLUCC 15-0152^T^), but only with a 85% ML bootstrap support. GUCC 21504 and GUCC 21505 formed an independent clade with MP and ML (68/90) supports and were close to *N.protearum* (CBS 111506^T^). GUCC 21506 and GUCC 21507 clustered with moderate and high supports (65/99/1: MP/ML/BI) and kept a very close relationship with *N.saprophytica* (CBS 115452) by credible statistic support (100/67/1: MP/ML/BI). DNA sequence differences between our strains and related species are listed in Table 2.

## Discussion

Hu et al. (2007) believed that pestalotiopsis-like fungi had different phenotypes in conidial morphology. Maharachchikumbura et al. (2014) summarised some stable characteristics for determining pestaloids, such as the length and width of conidia, length of the apical appendages, presence or absence of knobbed apices and the position of the apical appendage attached to the conidial body. However, as these characteristics were often similar or overlapped, sequence data are crucial for the identification of pestalotioid, and as well as for the introduction of new species (Norphanphoun et al. 2019).

In this study, we describe two new species as *Neopestalotiopsisrhapidis* and *N.rhododendri*. The species were distinct from extant *Neopestalotiopsis* species, based on morphological and phylogenetic analyses. However, the statistical support of main nodes for the genus were very low (Fig. 4). The reason might be that the reference sequences we used were short, including the short *tef1* and *tub2* sequences (Ran et al. 2017). Longer sequences with more informative data are needed to solve this problem. Furthermore, our study also found that the evolutionary relationships amongst species of *Neopestalotiopsis* are unstable (Maharachchikumbura et al. 2014, Jiang et al. 2018, Kumar et al. 2019, Tsai et al. 2020). Therefore, other genes are needed to distinguish the inter-species relationships in *Neopestalotiopsis* (Maharachchikumbura et al. 2014, Kumar et al. 2019, Norphanphoun et al. 2019).

Several indicators could be used in the classification of *Neopestalotiopsis* in this study, such as the size of conidia and the number and length of appendages (Maharachchikumbura et al. 2014, Freitas et al. 2019, Kumar et al. 2019). The differences in the colour of three median cells and the length of other cells, however, lacked significant variation to clearly distinguish the species of *Neopestalotiopsis*. Therefore, as the morphological identification alone cannot accurately identify the fungi of the genus *Neopestalotiopsis*, it must be combined with the phylogenetic tree (Liu et al. 2019, Norphanphoun et al. 2019, Tsai et al. 2020, Jiang et al. 2021).

## Supplementary Material

XML Treatment for Neopestalotiopsis rhapidis

XML Treatment for Neopestalotiopsis rhododendri

XML Treatment for Neopestalotiopsis saprophytica

## Figures and Tables

**Table 1.** The reference sequences used for phylogenetic analyses in this study with their GenBank accession numbers. (T) = ex-type strain.

Species name	Strain number	GenBank Accession numbers			Reference
		ITS	*tub2*	*tef1*	
* Neopestalotiopsis acrostichi *	MFLUCC 17-1754^T^	MK764272	MK764338	MK764316	Norphanphoun et al. (2019)
	MFLUCC 17-1755	MK764273	MK764339	MK764317	Norphanphoun et al. (2019)
* N. alpapicalis *	MFLUCC 17-2544	MK357772	MK463545	MK463547	Kumar et al. (2019)
	MFLUCC 17-2545	MK357773	MK463546	MK463548	Kumar et al. (2019)
* N. aotearoa *	CBS 367.54^T^	KM199369	KM199454	KM199526	Maharachchikumbura et al. (2014)
	HNPeHNLD2001	MT764947	MT796262	MT800516	Direct submission
* N. asiatica *	MFLUCC 12-0286^T^	JX398983	JX399018	JX399049	Maharachchikumbura et al. (2012)
* N. australis *	CBS 114159^T^	KM199348	KM199432	KM199537	Maharachchikumbura et al. (2014)
* N. brachiata *	MFLUCC 17-1555^T^	MK764274	MK764340	MK764318	Norphanphoun et al. (2019)
* N. brasiliensis *	COAD 2166^T^	MG686469	MG692400	MG692402	Bezerra et al. (2018)
* N. chiangmaiensis *	MFLUCC 18-0113^T^	-	MH412725	MH388404	Tibpromma et al. (2018)
* N. chrysea *	MFLUCC 12-0261^T^	JX398985	JX399020	JX399051	Maharachchikumbura et al. (2012)
* N. clavispora *	MFLUCC 12-0281^T^	JX398979	JX399014	JX399045	Maharachchikumbura et al. (2012)
	CBS 447.73	KM199374	KM199443	KM199539	Maharachchikumbura et al. (2014)
* N. cocoes *	MFLUCC 15-0152^T^	KX789687	-	KX789689	Hyde et al. (2016)
* N. coffea-arabicae *	HGUP4015^T^	KF412647	KF412641	KF412644	Song et al. (2013)
* N. cubana *	CBS 600.96^T^	KM199347	KM199438	KM199521	Maharachchikumbura et al. (2014)
* N. dendrobii *	MFLUCC 14-0106^T^	MK993571	MK975835	MK975829	Ma et al. (2019)
	MFLUCC 14-0132	MK993572	-	MK975830	Ma et al. (2019)
* N. egyptiaca *	COAD 2167	MG686470	MG692401	MG692403	Silva et al. (2018)
* N. ellipsospora *	MFLUCC 12-0284	JX398981	JX399015	JX399046	Maharachchikumbura et al. (2012)
	MFLUCC 12-0283^T^	JX398980	JX399016	JX399047	Maharachchikumbura et al. (2012)
* N. eucalypticola *	CBS 264.37^T^	KM199376	KM199431	KM199551	Maharachchikumbura et al. (2014)
* N. foedans *	CGMCC3.9178	JX398989	JX399024	JX399055	Maharachchikumbura et al. (2014)
	CGMCC3.9123^T^	JX398987	JX399022	JX399053	Maharachchikumbura et al. (2014)
* N. formicarum *	CBS 362.72^T^	KM199358	KM199455	KM199517	Maharachchikumbura et al. (2014)
	CBS 115.83	KM199344	KM199444	KM199519	Maharachchikumbura et al. (2014)
* N. honoluluana *	CBS 111535	KM199363	KM199461	KM199546	Maharachchikumbura et al. (2014)
	CBS 114495^T^	KM199364	KM199457	KM199548	Maharachchikumbura et al. (2014)
* N. iranensis *	CBS 137768^T^	KM074048	KM074057	KM074051	Ayoubi and Soleimani (2016)
* N. javaensis *	CBS 257.31^T^	KM199357	KM199437	KM199543	Maharachchikumbura et al. (2014)
	MFLUCC 12-0594	KX816905	KX816933	KX816874	Maharachchikumbura et al. (2014)
* N. keteleeria *	MFLUCC 13-0915	KJ503820	KJ503821	KJ503822	Song et al. (2014)
* N. macadamiae *	BRIP 63737c^T^	KX186604	KX186654	KX186627	Akinsanmi et al. (2017)
	BRIP 63757a	KX186592	KX186674	KX186647	Akinsanmi et al. (2017)
* N. magna *	MFLUCC 12-0652^T^	KF582795	KF582793	KF582791	Maharachchikumbura et al. (2014)
* N. mesopotamica *	CBS 336.86^T^	KM199362	KM199441	KM199555	Maharachchikumbura et al. (2014)
	CBS 299.74	KM199361	KM199435	KM199541	Maharachchikumbura et al. (2014)
* N. musae *	MFLUCC 15-0776^T^	KX789683	KX789686	KX789685	Hyde et al. (2016)
* N. natalensis *	CBS 138.41^T^	KM199377	KM199466	KM199552	Maharachchikumbura et al. (2014)
* N. nebuloides *	BRIP 66617^T^	MK966338	MK977632	MK977633	Crous et al. (2020)
* N. pandanicola *	KUMCC 17-0175^T^	-	MH412720	MH388389	Tibpromma et al. (2018)
* N. pernambucana *	URM7148	-	-	KU306739	Silvério et al. (2016)
* N. petila *	MFLUCC 17-1737^T^	MK764275	MK764341	MK764319	Norphanphoun et al. (2019)
	MFLUCC 17-1738	MK764276	MK764342	MK764320	Norphanphoun et al. (2019)
* N. phangngaensis *	MFLUCC 18-0119^T^	MH388354	MH412721	MH388390	Tibpromma et al. (2018)
	MFLUCC 19-2741	-	MW148259	MW192200	Direct submission
* N. piceana *	CBS 394.48^T^	KM199368	KM199453	KM199527	Maharachchikumbura et al. (2014)
	CBS 254.32	KM199372	KM199452	KM199529	Maharachchikumbura et al. (2014)
* N. protearum *	CBS 114178^T^	JN712498	KM199463	KM199542	Maharachchikumbura et al. (2014)
	CBS 111506	MH553959	MH554618	MH554377	Liu et al. (2019)
* N. rhapidis *	GUCC 21501	MW931620	MW980441	MW980442	This study
* N. rhizophorae *	MFLUCC 17-1551^T^	MK764277	MK764343	MK764321	Norphanphoun et al. (2019)
	MFLUCC 17-1550	MK764278	MK764344	MK764322	Norphanphoun et al. (2019)
* N. rhododendri *	GUCC 21504	MW979577	MW980443	MW980444	This study
	GUCC 21505	MW979576	MW980445	MW980446	This study
* N. rosae *	CBS 101057^T^	KM199359	KM199429	KM199523	Maharachchikumbura et al. (2014)
	CBS 124745	KM199360	KM199430	KM199524	Maharachchikumbura et al. (2014)
* N. rosicola *	CFCC 51992	KY885239	KY885245	KY885243	Jiang et al. (2018)
	CFCC 51993	KY885240	KY885246	KY885244	Jiang et al. (2018)
* N. samarangensis *	CBS 115451	KM199365	KM199447	KM199556	Maharachchikumbura et al. (2014)
	SS010	JQ968609	JQ968610	JQ968611	Direct Submission
* N. saprophytica *	CBS 115452	KM199345	KM199433	KM199538	Maharachchikumbura et al. (2014)
	GUCC 21506	MW979578	MW980447	MW980449	This study
	GUCC 21507	MW979579	MW980448	MW980450	This study
* N. sichuanensis *	CFCC 54338	MW166231	MW218524	MW199750	Jiang et al. (2021)
	SM15-1C	MW166232	MW218525	MW199751	Jiang et al. (2021)
* N. sonneratae *	MFLUCC 17-1744^T^	MK764279	MK764345	MK764323	Norphanphoun et al. (2019)
	MFLUCC 17-1745	MK764280	MK764346	MK764324	Norphanphoun et al. (2019)
*Neopestalotiopsis* sp.1	CFCC 54337	MW166233	MW218526	MW199752	Jiang et al. (2021)
	ZX12-1	MW166234	MW218527	MW199753	Jiang et al. (2021)
*Neopestalotiopsis* sp.2	CFCC 54340	MW166235	MW218528	MW199754	Jiang et al. (2021)
	ZX22B	MW166236	MW218529	MW199755	Jiang et al. (2021)
*Neopestalotiopsis* sp. nov.	GUCC 210001	MW930715	MZ683390	MZ683389	Direct Submission
*Neopestalotiopsis* sp. nov.	GUCC 210002	MW930716	MZ683391	MZ203452	Direct Submission
*Neopestalotiopsis* sp. nov.	GUCC 210003	MW936717	MZ683392	MZ540914	Direct Submission
* N. steyaertii *	IMI 192475^T^	KF582796	KF582794	KF582792	Jiang et al. (2021)
* N. surinamensis *	CBS 450.74^T^	KM199351	KM199465	KM199518	Maharachchikumbura et al. (2014)
	CBS 111494	-	KM199462	KM199530	Maharachchikumbura et al. (2014)
* N. thailandica *	MFLUCC 17-1730^T^	MK764281	MK764347	MK764325	Norphanphoun et al. (2019)
	MFLUCC 17-1731	MK764282	MK764348	MK764326	Norphanphoun et al. (2019)
* N. umbrinospora *	MFLUCC 12-0285^T^	JX398984	JX399019	JX399050	Maharachchikumbura et al. (2014)
* N. vitis *	MFLUCC 17-1108	MG807045	MG859849	MG859769	Jayawardena et al. (2016)
* N. zimbabwana *	CBS 111495^T^	-	KM199456	KM199545	Maharachchikumbura et al. (2014)
* Pestalotiopsis diversiseta *	MFLUCC 12-0287^T^	JX399009	JX399040	JX399073	Maharachchikumbura et al. (2012)
* P. trachicarpicola *	OP068^T^	JQ845947	JQ845945	JQ845946	Zhang et al. (2012)

**Table 2.** DNA sequence differences of the three gene regions between our strains and related species.

Species	Strain number	*tef1*	*tub2*	ITS
					(characters: 1-606)	(characters: 607-1443)	(characters: 1444-2052)
		* N. rhapidis *	GUCC 21501	0	0	0
* N. cocoes *	MFLUCC 15-0152^T^	27 (gaps: 2)	-	3 (gaps: 3)
Species	Strain number	*tef1*	*tub2*	ITS
					(characters: 1-606)	(characters: 607-1443)	(characters: 1444-2052)
		* N. saprophytica *	GUCC 21506	0	0	0
	GUCC 21507	0	0	0
* N. dendrobii *	MFLUCC 14-0106^T^	5 (gaps: 3)	4 (gap: 1)	1 (gap: 0)
* N. saprophytica *	CBS 115452	9 (gaps: 3)	1 (gap: 0)	1 (gap: 1)
Species	Strain number	*tef1*	*tub2*	ITS
					(characters: 1-606)	(characters: 607-1443)	(characters: 1444-2052)
		* N. rhododendri *	GUCC 21504	0	0	0
	GUCC 21505	9 (gap: 0)	2 (gap: 0)	7 (gap: 1)
* N. protearum *	CBS 114178^T^	12 (gaps: 6)	3 (gap: 0)	9 (gaps: 2)
